# Structural basis for the membrane association of ankyrinG via palmitoylation

**DOI:** 10.1038/srep23981

**Published:** 2016-04-05

**Authors:** Yuichiro Fujiwara, Hiroko X. Kondo, Matsuyuki Shirota, Megumi Kobayashi, Kohei Takeshita, Atsushi Nakagawa, Yasushi Okamura, Kengo Kinoshita

**Affiliations:** 1 Integrative Physiology, Department of Physiology, Graduate School of Medicine, Osaka University, Suita, JAPAN; 2 Systems Bioinformatics, Graduate School of Information Sciences, Tohoku University, Sendai, JAPAN; 3 Systems Bioinformatics, Tohoku Medical Megabank Organization, Tohoku University, Sendai, JAPAN; 4 Institute of Development, Aging and Cancer, Tohoku University, Sendai, Japan; 5 United Centers for Advanced Research and Translational Medicine, Graduate School of Medicine, Tohoku University, Sendai, JAPAN; 6 Supramolecular Crystallography, Research Center for Structural and Functional Proteomics, Institute for Protein Research, Osaka University, Suita, JAPAN; 7 Graduate School of Frontier Biosciences, Osaka University, Suita, JAPAN; 8 Graduate School of Information Sciences, Hiroshima City University, Hiroshima, JAPAN

## Abstract

By clustering various ion channels and transporters, ankyrin-G (AnkG) configures the membrane-excitation platforms in neurons and cardiomyocytes. AnkG itself localizes to specific areas on the plasma membrane via s-palmitoylation of Cys. However, the structural mechanism by which AnkG anchors to the membrane is not understood. In this study, we solved the crystal structures of the reduced and oxidized forms of the AnkG s-palmitoylation domain and used multiple long-term coarse-grained molecular dynamics simulations to analyze their membrane association. Here we report that the membrane anchoring of AnkG was facilitated by s-palmitoylation, defining a stable binding interface on the lipid membrane, and that AnkG without s-palmitoylation also preferred to stay near the membrane but did not have a unique binding interface. This suggests that AnkG in the juxtamembrane region is primed to accept lipid modification at Cys, and once that happens AnkG constitutes a rigid structural base upon which a membrane-excitation platform can be assembled.

The electrical activity in the heart and brain is based on the flux of ions across the cell membrane and is mediated by the activities of ion channels, pumps and exchangers. These ion transporting molecules are not distributed randomly in the plasma membrane, but are constrained to specific areas defined by scaffold proteins that bind multiple members of a signaling pathway, tethering them into complexes. One of the major extrasynaptic scaffold proteins is Ankyrin-G (AnkG, encoded by ANK3 gene), which localizes various ion channels, adhesion molecules and receptors by linking to the spectrin cytoskeleton^1^. AnkG has been detected in the axon initial segment^2^ and in nodes of Ranvier^3^, where it tethers voltage-gated ion channels^4^, as well as in the rod outer segment of the retina^5^, costameres in skeletal muscle^6^ and cardiac intercalated discs^7^ where it mediates formation of signaling complexes. Disorder of AnkG-mediated channel clustering within the heart induces arrhythmias such as Brugada syndrome^8^ and sinus node dysfunction^7^, while AnkG in the brain appears to be involved in bipolar disorder^9^ and autism spectrum disorder^10^.

The sites at which AnkG locates are not dependent on the distribution of its binding partners^11^,^12^,^13^, but on its palmitoylation^14^,^15^, as is also the case for other scaffold proteins^16^. Palmitoylation is the reversible covalent attachment of fatty acids to Cys, which enhances the hydrophobicity of proteins and appears to play a significant role in the subcellular trafficking of proteins to membrane compartments. Palmitoylation at the Cys (s-palmitoylation) in AnkG was recently shown to be a key determinant for its targeting to epithelial lateral membranes and neuronal axon initial segments^15^. Palmitoyltransferase DHHC5/8 is required for AnkG palmitoylation and for its localization at the lateral membranes of MDCK cells^14^. Although s-palmitoylation of synaptic scaffold proteins and their accompanying regulation (e.g., redox-regulation and s-nitrosylation) have been studied extensively with respect regulation of neurotransmission^17^,^18^,^19^,^20^,^21^, the study of ankyrin palmitoylation has only just begun and much remains unknown.

Ankyrin family proteins, ankyrin-R (AnkR, encoded by ANK1 gene), ankyrin-B (AnkB, encoded by ANK2 gene) and AnkG, all consist of four functional domains: a membrane-binding domain, a spectrin binding domain, a death domain that binds to proteins involved in apoptosis, and a variable C-terminal regulatory domain^1^ (Fig. 1A). The membrane-binding domain consists of 24 tandem ankyrin repeats that bind to various types of ion channels and cell adhesion proteins (Fig. 1A). This domain also contains a conserved Cys within a loop structure between the 1st and 2nd repeats, which is reported to be the s-palmitoylation site in AnkG^15^ (Fig. 1A). There have been a few studies of the structural biology of the membrane-binding domain (e.g., the latter half segment of the membrane-binding domain of AnkR^22^ and the structure of the AnkB membrane-binding domain in complex with regulatory peptides^23^) but no studies of the structure of AnkG. Particularly desirable, given its impact on the distribution of signaling complexes, is a better understanding of the atomic structure of AnkG palmitoylation. Association of proteins with the plasma membrane via lipid modification has been studied using various cell biological assays, but the structural basis for the interaction remains largely unknown.

In the present study, we solved the crystal structure of the s-palmitoylation region of the AnkG membrane-binding domain and performed a molecular dynamics (MD) simulation of the membrane association of AnkG. Multiple long-term simulations shed light on the features of protein anchoring to the lipid membrane via palmitoylation.

## Results

### Structural conformation of the AnkG palmitoylation domain

**Figure 1:**
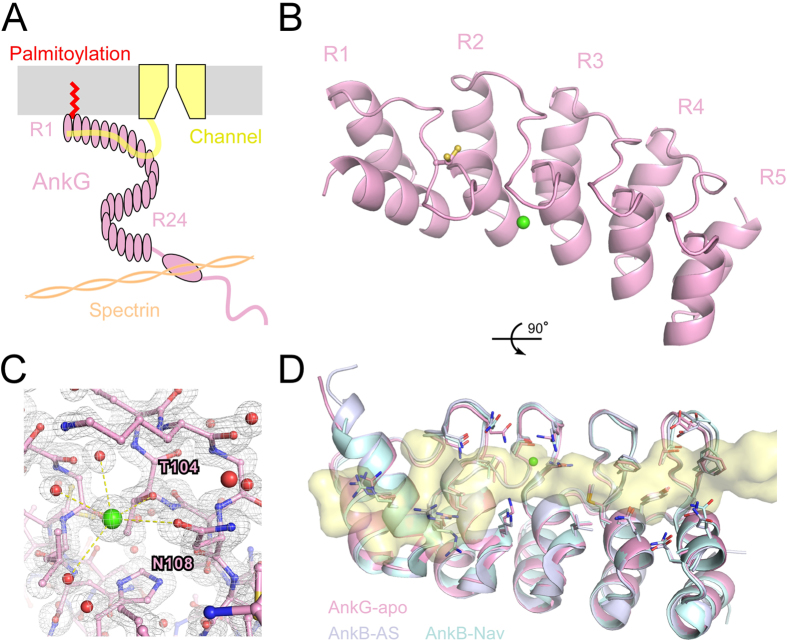
Crystal structure of the palmitoylation domain of AnkG. (**A**) Schematic drawing of the membrane excitation platform. AnkG binds to the plasma membrane via s-palmitoylation, tethering ion channels and the cytoskeleton to the complex. (**B**) Crystal structure of the reduced form of AnkG (R1-R5). The dual conformation of the Cys side-chain is shown as sticks. Ca^2+^ is shown as a green sphere. Orientations of the repeats (R1-R5) are indicated. (**C**) Structure (stick models) and 2Fo-Fc maps of Ca^2+^ (green sphere) and its surroundings. The maps are contoured at 1.5 σ. Red balls depict oxygen atoms of water molecules. Yellow dashed lines depict polar contacts. (**D**) Structural comparison of AnkG and AnkB. AnkG-apo depicts the reduced form of AnkG. AnkB-AS depicts AnkB in complex with the auto-inhibitory segment (AS) of AnkR (pdb code # 4RLV)^23^, though the AS peptide is not shown. AnkB-Nav depicts AnkB in complex with the peptide fragment of the voltage-gated Na^+^ channel (Nav) (pdb code # 4RLY)^23^. Side-chains that interact with the regulatory peptide are shown as sticks. The yellow surface depicts the structure of the Nav peptide.

The 33 amino acid residues that comprise each of the 24 ankyrin repeats in the AnkG membrane binding domain are aligned with great regularity (Fig. 1A and Supplementary Fig. S1). An exceptionally short sequence links the 5th repeat (R5) and R6, while an extra sequence exists between R6 and R7, and this pattern is well conserved among all three known ankyrins (AnkR, AnkB and AnkG) (Supplementary Fig. S1). The membrane-binding domain also contains a conserved cysteine residue (Cys70) within the loop structure between R1 and R2 (Supplementary Fig. S1), which is reportedly the s-palmitoylation site in AnkG. Ankyrin repeat domains are widely distributed among many types of proteins, and a multiple sequence alignment focusing on the R1-R2 region of the ankyrin repeat domain is shown in Supplementary Fig. S2. Particularly interesting to us was the fact that the Cys residue within the loop structure between R1 and R2 is rare, making lipid anchoring at the head-section of the ankyrin repeat domain unique to AnkR, AnkB and AnkG.

We therefore expressed, purified and crystallized proteins consisting of the head-section of the membrane-binding domain (R1-R5) of rat AnkG. Screening yielded two AnkG (R1-R5) crystal forms, orthorhombic P2_1_2_1_2 and monoclinic C121 (Supplementary Fig. S3A,B), which diffracted synchrotron X-rays to 1.62 Å and 1.83 Å, respectively (Table 1). The structure of the P2_1_2_1_2 crystal was determined by molecular replacement using the R13-R17 segment of AnkR (PDB code #1N11)^22^, and an initial structural model was built and refined to an acceptable level (R/Rfree = 16.5/20.8%) (Table 1). Structural determination of the C121 crystal was achieved through molecular replacement using the initial AnkG (R1-R5) crystal structure, after which the resultant structure was refined (Table 1).

The structure of the P2_1_2_1_2 crystal shows the typical ankyrin-repeat structure in which each repeat consists of 33 amino acid residues forming two anti-parallel α-helices and a loop ending in a β-hairpin (Fig. 1B). Cys70 is exposed on the protein surface and shows some side-chain flexibility observed as a dual-conformation (Fig. 1B), suggesting an s-acylation-prone structure. The electron density of an ion was observed on the inner side of the finger loop of R2, and it is thought to be Ca^2+^ from the crystallization buffer (Fig. 1B,C). The Ca^2+^ forms hydrogen bonds with water molecules and with T104 and N108 (Fig. 1C). In AnkB, these two residues are reportedly essential for recognition of regulatory peptides^23^. The AnkG structure was compared to the structures of AnkB in complex with its regulatory peptides: the auto-inhibitory segment (AS) and the voltage-gated Na^+^ channel (Nav) (Fig. 1D). The AnkG (R1-R5) structure aligns well with the first 5 ankyrin repeats of the complexed AnkB, with overall Cα-r.s.m.d.s of 0.795 Å (AnkG vs. AnkB-Nav) and 0.705 Å (AnkG vs. AnkB-AS), respectively (Fig. 1D). The loop structure showed local variation between AnkG and AnkB at the initial segment of the R2 loop (Cα-r.s.m.d.s of 2.108 Å, AnkG vs. AnkB-Nav; 2.228 Å, AnkG vs. AnkB-AS) and at the finger loops of R4 (Cα-r.s.m.d.s of 1.582 Å, AnkG vs. AnkB-Nav; 0.646 Å, AnkG vs. AnkB-AS); the association with the function was not solved.

**Figure 2:**
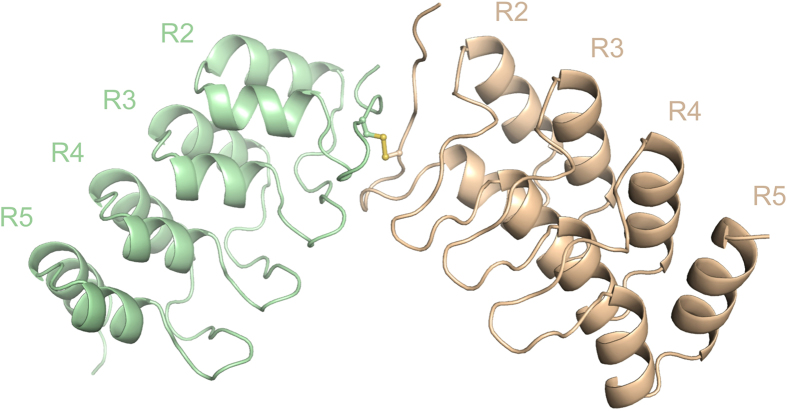
Crystal structure of the oxidized from of AnkG. Dimerized structure of AnkG. Sticks depict the disulfide bond between the two molecules. Electron density maps around C70 are shown in the Supplementary Fig. S4A.

The C121 crystal forms an oxidized dimer in which two ankyrin molecules are linked by a disulfide bond between the two Cys70 residues (Fig. 2). The R1 structures are unfolded in both molecules (Fig. 2), while the R2-R5 structures are very similar to the reduced form (Supplementary Fig. S3C,D). Ca^2+^ was not contained in the oxidized dimer (Fig. 2). Analytical gel filtration chromatography of AnkG showed that the two AnkG forms (oxidized/reduced) are switchable in solution (Supplementary Fig. S4B).

Although the AnkG structure is an apo-peptide, the arrangement of the side-chains in the inner groove, which interact with the regulatory peptides, show high similarity to those in complexed AnkB (Fig. 1D). The finger loop structure of R2 in AnkG, the Ca^2+^ binding pocket, also remained unaltered regardless of the redox condition (Supplementary Fig. S3C). We hence speculate that AnkB/G binds their binding partners with the lack of induced-fit of the structure.

### MD simulations of the AnkG membrane interaction

**Figure 3:**
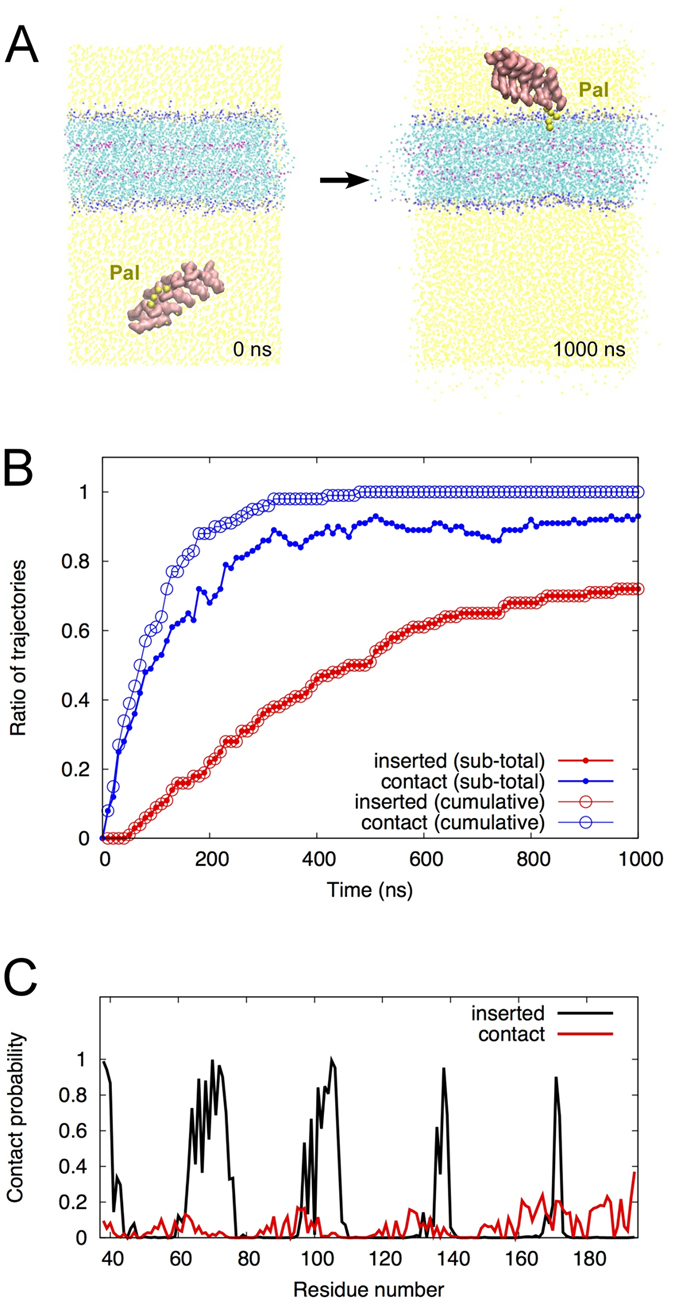
MD simulation of palmitoylated AnkG. (**A**) Long-term (1 μs) coarse-grained MD simulation of a palmitoylated AnkG (R1-R5)-membrane system. Representative initial (left) and final (right) orientations of AnkG are shown. (**B**) The time course of proximities between AnkG and the lipid membrane was analyzed as two kinds of events: “contact” and “contact after insertion; inserted.” (The definition of “contact” and “insertion” are provided in the Methods section.) For “contact,” cumulative and subtotals of the membrane contacts were analyzed for all 100 trials (blue lines), where subtotal is the ratio of trajectories in which the “contact” events were observed during the prior 10 ns. For “inserted,” the ratios of “contact” trajectories undergoing “insertion” events were analyzed for the 72 trajectories in which insertion was observed (red lines). The cumulative and subtotal curves overlap (red lines). (**C**) Contact probability for the interaction between the lipid membrane and each residue. Details of the calculation of contact probability are provided in the Methods section. The black and red lines represent the respective contact probabilities calculated for the snapshots after and before the “insertion” event.

The crystal structure alone does not provide a clear understanding of the mechanism by which AnkG associates with the membrane in terms of its dynamics or the precise structure of the interface. Therefore, to analyze the behavior of AnkG in the vicinity of the plasma membrane, we performed MD simulations using three AnkG-membrane systems. We used coarse-grained MD simulations to statistically evaluate the contribution of palmitoylation to the membrane association. Coarse-grained simulation enables us to perform long-term simulations at a lower computational cost than all-atom simulations because of fewer degrees of freedom. We first set up a simulation system involving an s-palmitoylated AnkG (R1-R5) and a lipid bilayer. The distance along the z-axis (difference between the z coordinates) of the centers of mass of the membrane and protein was 70 Å (see the Methods section for simulation details). One hundred runs of a 1-μs simulation were carried out using different initial orientations of the protein, and the numbers of association events observed in these simulations were counted (Fig. 3B). The approach of AnkG to the lipid membrane was observed for all trajectories (Fig. 3B, blue cumulative line with open circles), but the ratio of trajectories in which the “contact” events were observed during the prior 10 ns (called as subtotal hereafter) was below 1.0, indicating that contacts were lost with some trajectories (Fig. 3B, blue subtotal line with filled circles). In 72 of the 100 trials, the palmitoylated AnkG was irreversibly anchored to the membrane by insertion of the acyl chain at Cys70 (Fig. 3B, red lines, and Fig. S4A), but usually the palmitoylated AnkG made and lost contact with the membrane before insertion of the s-palmitoyl group (Supplementary Fig. S5A). During the simulation, the free exposure of the palmitate moiety was in a short period of time, and in some case, it was sticking to the AnkG protein surface against the hydrophobic mismatch. Note that we defined each snapshot as a “contact” if the minimum distance between the membrane atoms and AnkG atoms was less than 6 Å, and a trajectory with an insertion event of the s-palmitoylated AnkG was regarded as an inserted trajectory. See Materials and Methods for more details of the analysis.

The proximity of AnkG to the membrane was also analyzed by calculating the “contact probability” for each residue (Fig. 3C). The contact probabilities for the snapshots before the insertion event (Fig. 3C, red line) were low (<~0.2) and distributed randomly over residues located on the protein surface (Fig. 3C and Supplementary Fig. S6). This suggests AnkG has no specific interaction with the membrane before anchoring. On the other hand, once a palmitoylation anchor was inserted into a membrane, only the specific residues contributing to the interaction surface had high contact probabilities, which is indicative of the importance of the palmitoylation of AnkG for stable contact with the membrane.

**Figure 4:**
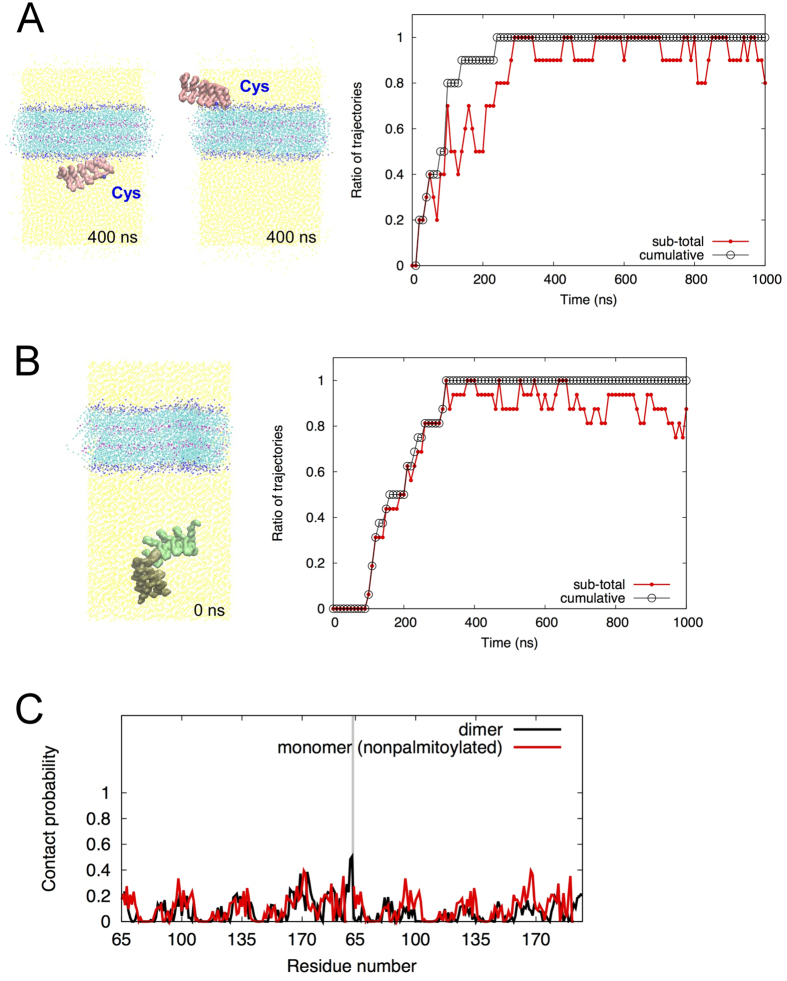
MD simulation of non-palmitoylated AnkG. (**A**) Long-term (1 μs) coarse-grained MD simulation of nonpalmitoylated AnkG (R1-R5). The left panels show representative orientations observed during the simulations. The time course of the proximities of the AnkG to the lipid bilayer is shown in the right panel. The subtotal represents the ratio of trajectories in which “contact” events were observed during the prior 10 ns. (**B**) Long-term (1 μs) coarse-grained MD simulation of dimeric AnkG. The left panel is a representative initial structure of the system. The right panel represents the time course of the observed protein-membrane association events in 16 trials (same as shown in (**A**)). (**C**) Contact probabilities for the interaction between the lipid membrane and each residue. Details of the calculation of contact probabilities are provided in the Methods section. The black and red lines represent the contact probabilities for the AnkG dimer and nonpalmitoylated AnkG simulations, respectively. The perpendicular gray line shows the terminal of the former monomeric subunit. The residue numbers of the nonpalmitoylated AnkG are duplicated corresponding to the two monomeric subunits of the AnkG dimer.

In 10 trials, similar contacts between non-palmitoylated AnkG and a lipid bilayer were observed. The time course of contact events is summarized in Fig. 4A. In all 10 trajectories, a non-palmitoylated AnkG moved to the membrane surface and made and lost contact with the membrane in various styles with different interfaces (Fig. 4A). But without the palmitic acyl chain, the AnkG could not anchor to the membrane or retain a specific binding interface (Fig. 4A).

Dimeric AnkG with a disulfide bond at Cys70 was also analyzed with a system expanded along the z-axis in consideration of the doubled size of the AnkG molecule (Fig. 4B). In 16 trials the dimeric AnkG moved to the membrane surface and made and lost contact in a manner similar to non-palmitoylated AnkG (Fig. 4A). Contact probabilities for interaction between each residue and the membrane in both non-palmitoylated and dimeric AnkG were low for all residues, and showed a pattern similar to that observed with contact of palmitoylated AnkG (Fig. 4C), indicating non-specific contacts of the protein with the membrane. Thus s-palmitoylation was the key determinant of AnkG anchorage to the membrane via a defined binding interface, though AnkG showed a tendency to make contact with the membrane regardless of the palmitoylation.

**Figure 5:**
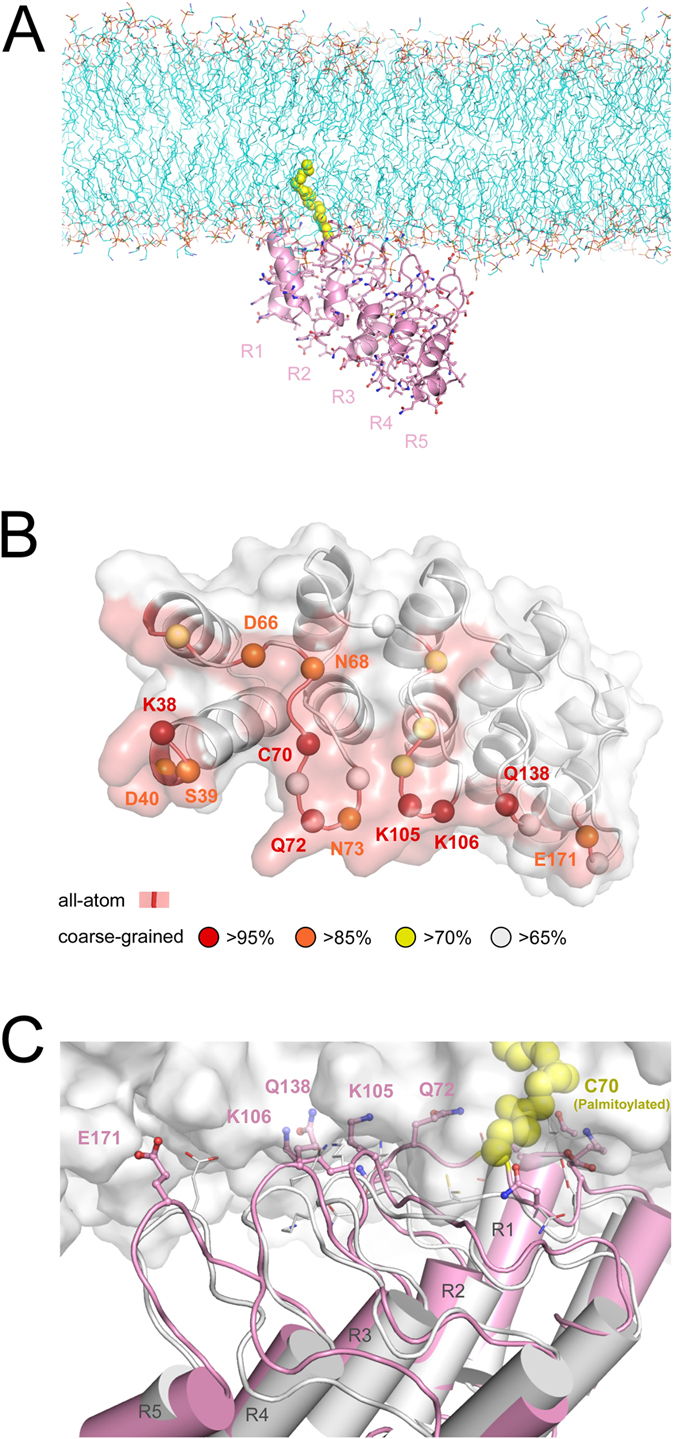
The membrane binding conformation of palmitoylated AnkG. (**A**) Overall orientation of the fine-grained (all-atom) structure of the membrane-adherent form of AnkG. Cyan line models depict membrane lipids, and a string of yellow spheres depicts an acyl chain of the palmitoylated Cys70. (**B**) Mapping of the contribution to membrane binding. Residues that showed high contact probability in the coarse-grained MD simulation of the palmitoylated AnkG after insertion are indicated as colored spheres. Residues that make contact with the membrane phospholipids in the all-atom simulation are indicated as red surfaces. (**C**) Structural comparison between the crystal structure (white cartoon) and the fine-grained structural model (pink cartoon). Side-chains of the residues indicating high contact probabilities are also shown as stick/line models. A white surface indicates the surface of the membrane phospholipids.

The structure of the membrane-anchored palmitoylated AnkG (R1-R5) was examined in more detail by running a full-atom MD simulation. A major coarse-grained conformation was converted to a fine-grained (all-atom) structure using a modified backward.py script^24^, and the side-chain conformations were relaxed in a 5-ns simulation with position restraints on the backbone atoms (Fig. 5A). To elucidate the interactions, side-chains contributing to the membrane binding were mapped on the structure, and several important residues in the loop fingers were highlighted (Fig. 5B). The overall orientation (Fig. 5A) and binding interface (Fig. 5B) indicate that the structure around the palmitoylated Cys and loop fingers of R1-R3 comprise the binding interface. This suggests the membrane association via s-palmitoylation is almost accomplished with the head region of the ankyrin repeats (R1-R5). The loop finger structures and side-chain orientation exhibit the flexibility to interact with the membrane phospholipid head groups, which was not apparent from the crystal structure (Fig. 5C). Thus AnkG orients to the membrane due to the hydrophobic insertion of the palmitoyl acyl chain into the lipid core and to the hydrophilic interactions around the loop fingers at the membrane boundary.

## Discussion

In this study, we used X-ray crystallography and MD simulation to analyze the association of AnkG with a membrane via s-palmitoylation. Our crystal structure analysis highlighted some of the structural features of AnkG and its regulation. First, the AnkG structure is an apo-peptide, but shows high similarity to the peptide-binding structures of AnkB down to the side-chain arrangement in the inner groove (Fig. 1D). This is estimated to be the lack of induced-fit to peptides, suggesting low ligand-specificity, which may confer the capacity to accommodate target proteins with diverse sequences^25^. Second, the AnkG structure includes Ca^2+^ that interacts with T104 and N108 (Fig. 1C), two residues that are reportedly essential for recognition of Nav and AS, two AnkB regulatory peptides^23^. This suggests Ca^2+^ may serve as a regulatory molecule competing with Nav and AS. This is noteworthy as Ca^2+^ channels are concentrated in the axon initial segment (AIS)^26^, and it has been hypothesized that Ca^2+^ serves as a trigger for the activity-dependent relocation of AIS components, including AnkG and Nav^27^,^28^. Third, the two forms of the AnkG structure, oxidized and reduced, are switchable (Fig. 2 and Supplementary Fig. S4B). Several examples of redox-regulated scaffold proteins have been reported. For example, PICK1, which controls the trafficking of various channels and receptors, switches to a dimeric form through oxidation, thereby abolishing its membrane binding capacity^21^. Although the redox-regulation of AnkG has not yet been demonstrated, its structure may provide clues to ankyrin’s redox-dependent regulation in physiological and pathological situations such as oxidative stress and ischemia.

Coarse-grained MD simulations showed that AnkG tends to move to the lipid membrane irrespective of its palmitoylation (Figs 3 and 4). AnkG has no specific stable contacts but stayed near the membrane surface while presenting different interfaces (Figs 3 and 4 and supplementary Fig. S6). The palmitoyltransferases DHHC5 and DHHC8 assume vital roles in AnkG palmitoylation and thus its localization at the plasma membrane^14^. DHHCs are membrane proteins with an active cytoplasmic catalytic domain, and its catalytic center is located only about 20 residues away from the transmembrane segment. This implies they are able to palmitoylate proteins in the juxtramembrane region, which also suggests that the initial arrangement of our MD simulation with palmitoylated AnkG fits the distance from the membrane. Since the existence of free-floating palmitoylated AnkG remains incompletely understood, the process of membrane insertion is speculative. However, our simulation analysis of the palmitoylated AnkG (Fig. 3) yielded valuable information: (1) irreversibility of the membrane insertion of palmitoylated AnkG and (2) highlight of the membrane-interacting residues of palmitoylated AnkG when inserted. In addition, the membrane preference of AnkG indicated by MD simulations (Figs 3 and 4) may increase the likelihood of its palmitoylation by the DHHC membrane proteins. The protein palmitoylation event shows variable reversibility, i.e. s-palmitoylation appears rapidly reversible in cells^18^,^29^,^30^, while irreversible palmitoylations are also reported^31^,^32^. Palmitoylation of AnkR reportedly shows reversible nature^33^. Although the present study did not assess the reversibility of AnkG palmitoylation, regardless of any potential reversible nature, AnkG may be primed to accept the Cys modification in the juxtamembrane region; once that happens, AnkG becomes a rigid structural base for a membrane-excitation platform. The palmitoylation of another protein, PSD-95, reportedly arises on the Golgi or plasma membrane, depending on the trafficking of DHHC proteins, and plays important roles in the activity-dependent relocalization of AMPA receptors in the neuronal synapse^17^,^34^. In that context, the subcellular spatial and temporal distribution of AnkG palmitoylation merits further study.

MD simulations also present a membrane-adherent form of palmitoylated AnkG (Fig. 5). Although the structure contained only part of the AnkG molecule (Fig. 5A), it revealed some key residues clustered on the palmitoylation plane that comprised the binding interface (Fig. 5B), and also showed a dynamic interaction between side-chains and polar headgroups of the phospholipids (Fig. 5C), which could not be observed in the frozen crystal structure. Lys105 and Lys106 in the finger loop of R2 were highlighted as the major residues interacting with the membrane (Fig. 5B,C), and are also reportedly critical residues binding with Nav^35^. This is noteworthy as it suggests there may be collaboration/competition between membrane anchoring and Nav tethering within axon initial segments. To obtain an image of the full-repeat orientation of AnkG along the membrane, we tried to fit the structure of AnkB (R1-R24) onto the membrane-adherent AnkG form (R1-R5) but failed due to steric hindrance from the membrane in the tail region of the repeat (R18-R24). We speculate that to achieve an ideal balance, the ankyrin repeat structure is somewhat flexible, which has been proposed previously^36^,^37^. This appears particularly important in the R5-R6 region, perhaps due to the irregular amino acid sequence (Supplementary Fig. 1). In fact, a recently reported AnkB structure (R1-R9, PDB code: #4RLY) showed an inversion of the repeat structure at the connection between R5 and R6^23^.

A number of physiological functions derived from lipid modification of proteins have been proposed^38^. Understanding the structural mechanism by which these proteins associate with the membrane via lipid modification has grown increasingly important. The structural dynamics approach used in this study should be widely applicable to investigations of protein anchoring to lipid membranes. A uniform phospholipid (POPE) bilayer was used in our simulation system, while the plasma membrane consists of multiple components of lipids. Minor components of membrane lipids such as phosphoinositols and cholesterols may possibly affect the protein binding. If the membrane lipid composition in the AnkG anchoring region will become clear in the future, simulation analyses with mixed membrane lipids will generate further outcomes to understand the mechanism.

## Methods

### Molecular cloning

We used a rat AnkG clone (270 kDa) for all experiments in this study. DNA corresponding to the first five ankyrin repeats of the membrane binding domain (R1-R5: residues 38–200) was amplified by PCR and ligated into a pCold (Takara Bio Inc., Japan)-derived vector, in which an original cleavage site for the Factor Xa protease was replaced with a Tobacco Etch Virus (TEV) protease cleavage site. The resultant construct encodes a protein that has only two extra residues, Gly-Thr, at the N-terminus after the TEV cleavage.

### Protein expression and purification

AnkG (R1-R5) proteins were expressed in E.coli [BL21(DE3)pLysS] grown in 2YT media at 16 °C and induced with 0.4 mM IPTG for 20 h. Cells were harvested by centrifugation, after which the cell pellets were lysed by sonication in lysis buffer (10 mM K_2_HPO_4_, pH 7.3, 250 mM KCl, 1 mM EDTA, 5 mM β-ME, 1 mM PMSF), and the insoluble material was removed by centrifugation. The remaining soluble fraction, which contained His-tagged AnkG proteins, was applied to a 20 ml HisPrep FF (GE Healthcare Japan, Japan) nickel-charged column, and eluted using 500 mM imidazole on an ÄKTA-purifier system (GE Healthcare Japan, Japan). After desalting the imidazole, the His-tagged proteins were cleaved using TEV protease (~300 μM for 24 h at 4 °C). AnkG proteins were collected in the flow-through from the Hisprep nickel column and extended into a superdex200 gel-filtration column (GE Healthcare Japan, Japan). The purified proteins were then further concentrated through buffer exchange using an Amicon centrifugal filter (Merck Millipore Corp., USA) to 10 mg/mL [dissolved in 50 mM NaCl, 5 mM Tris-HCl (pH 7.5)], and were then used for the crystallization step.

### Crystallization, data collection and structure determination

AnkG (R1-R5) crystals were grown on crystal screening plates at 20 °C using sitting-drop vapor diffusion. Crystals of the reduced form of AnkG grew from 1:1 mixtures of protein solution and reservoir solution containing 50 mM CaCl_2_, 100 mM Bis-Tris (pH 7.0), 30% w/v PEG550 MME (Index #26, Hamptom Research, USA) (Fig. 1). Crystals of the oxidized form of AnkG grew from 1:1 mixtures of protein solution and reservoir solution containing 10 mM MgCl_2_-hexahydrate, 50 mM Tris-HCl (pH 7.5), 1.6 M (NH_4_)_2_SO_4_ (Natrix #26, Hamptom Research, USA) (Fig. 2). For data collection, the crystals were transferred to 20–25% PEG200 cryoprotectant and flash-cooled in liquid nitrogen. Data were collected at BL44XU SPring-8 (Hyogo, JAPAN), which is equipped with a MX-225HE CCD detector (Rayonix, USA) and is financially supported by Academia Sinica and the National Synchrotron Radiation Research Center (Taiwan, ROC). The crystal of the reduced form belonged to the P2_1_2_1_2 space group and diffracted X-rays to 1.62 Å, while the crystal of the oxidized form belonged to the C121 space group and diffracted X-rays to 1.83 Å.

All data were processed using HKL2000 (HKL Research Inc., USA). The primary structure of the reduced form of ANKG (R1-R5) was solved by molecular replacement with Phaser^39^ using the AnkR membrane binding domain structure as a template (1N11, residues 637–791), and an initial structural model was built using the program Arp/Warp^40^. The initial structure was then used as a template in the molecular replacement for determination of the oxidized structure, and a model was successfully built using Arp/Warp, despite the unfolded structure of the 1st repeat (Fig. 2A). The P2_1_2_1_2 space group asymmetric unit of the reduced form contained two molecules, while the C121 space group asymmetric unit of the oxidized form contained one dimeric molecule. Model buildings were done in Coot^41^. We were able to assemble 158 residues in each of the two chains of the reduced form, and 134 residues in each two chains in the structure of the oxidized form. All models were refined using REFMAC5^42^, and TLS parameters were included when the oxidized structure was refined (Table 1).

The structural comparison and superposition of AnkG (40–194) and AnkB (2030–2184; PDB code #4RLV and #4RLY) were carried out using lsqkab in the CCP4 suite, and the r.m.s.d. was calculated (Fig. 1D). To compare the fine-grained model structure with the crystal structure (reduced form), we superposed the helical body structures (two of the five anti-parallel α-helices) and determined the structural deviation of the loop fingers (Fig. 5). The structures of the reduced and oxidized form of AnkG were also compared in the range of residues 68–194 (Supplementary Fig. S3). The initial segment of R2 (residues 95–100), the finger loop of R4 (residues 168–175) and the finger loop of R2 (residues 100–110) were also analyzed for structural comparisons (Fig. 1D and Supplementary Fig. S3). Surface areas of the structure models were calculated using areaimol in the CCP4 suite (Supplementary Fig. S6).

### Gel-filtration analysis of the redox-dependent dimerization

The molecular weights of the purified oxidized/reduced proteins were determined as described previously^21^,^43^,^44^. Purified AnkG (R1-R5) proteins (0.5 mg/mL) were incubated first with 100 μM H_2_O_2_ for 1 h or 4 h at 4 °C, and then with 5 mM DTT for 1 h at 4 °C after the oxidation. Protein solutions (100 μL each) were then passed through a superdex200 gel-filtration column (GE Healthcare Japan, Japan) equilibrated with the loading buffer (250 mM KCl, 10 mM K_2_HPO_4_ [pH 7.3]) on an ÄKTA-purifier system (GE Healthcare Japan, Japan) at 4 °C and a flow rate of 0.5 ml min^−1^. Eluates were monitored at 280 nm, and the molecular weights and stoichiometry of the proteins were analyzed on the basis of the elusion peaks using six standard protein molecular mass markers (GE Healthcare Japan, Japan) (Fig. 2C).

### Molecular dynamics simulation

All MD simulations were performed using the GROMACS software package version 4.6.5^45^. We performed coarse-grained MD simulations of three systems, palmitoylated-AnkG, nonpalmitoylated-AnkG and AnkG dimer, using Martini force field^46^,^47^,^48^ (ver. 2.2 for amino acids and ver. 2.0 for lipids and ions) to analyze the behavior of AnkG around a membrane. Force field parameters for the s-palmitoylated Cys were created by replacing the Cys side-chain atom with the palmitoyl acyl chain. All three systems consisted of a protein (AnkG), a lipid bilayer composed of 410 1-palmitoyl–2-oleoyl-phosphatidylethanolamine (POPE) molecules, water molecules and ions. The sizes of the systems were approximately 11 × 11 × 17 nm^3^, 11 × 11 × 17 nm^3^ and 11 × 11 × 20 nm^3^, respectively. The periodic boundary condition was applied to all simulations. By changing the protein arrangement, we prepared 100, 10 and 16 initial structures for the palmitoylated-AnkG, nonpalmitoylated-AnkG and AnkG dimer systems, respectively. Coarse-grained protein structures were constructed based on the X-ray structures using the martinize.py script, and an elastic network model was combined with the Martini model to preserve their tertiary structures. The lower and upper elastic bond cut-off values were set to 0.5 and 0.9 nm, respectively, and the elastic bond force constant was set to 5000 kJ.mol^−1^.nm^−2^. We minimized the energy of these systems, equilibrated them with position restraints for the backbone atoms, and then performed 1-μs production runs with a time step of 25 fs in NPT ensemble (310 K, 1 bar) using the V-rescale thermostat^49^ and Berendsen barostat^50^. The electrostatic interactions were calculated using the particle mesh Ewald (PME) method^51^,^52^.

All trajectories were analyzed with an interval of 2 ns (i.e., 500 snapshots for each 1-μs simulation). We regarded a snapshot as a “contact” if the minimum distance between membrane atoms and AnkG atoms was less than 6 Å. An “Insertion event” was defined as the first time interaction between the membrane and Cys70 of AnkG was observed in three successive snapshots, and trajectories with an “insertion event” were regarded as “inserted.” Contact probability between the membrane and AnkG was defined as the probability of being located in the vicinity of the lipid bilayer, and the probability was calculated for each residue as the ratio of snapshots in which there was interaction between the residue and atoms in a lipid head-group; i.e., C1A, C1B, NH3 and PO4 in a Martini force field, during the trajectories after an “insertion event” or the first “contact” between AnkG and the membrane.

We also performed fine-grained (all-atom) MD simulations to analyze the interaction between the lipid bilayer and AnkG in the atomic level (Fig. 5). A typical conformation of AnkG anchoring to the POPE membrane was selected and solvated with ~3.5 k water molecules and ~150 mM NaCl in a rectangular box. The simulations were done using a periodic boundary condition, and the electrostatic interactions were treated using the PME method. CHARMM36 force field^53^ was applied to the amino acids, lipid molecules and ions, and the TIP3P model^54^ was applied to the water molecules. Parameters for the s-palmitoylated Cys were created by combining the force field parameters of the Cys residue in CHARMM36 with those of the palmitoyl acyl chain in the lipids. All bonds were constrained by the LINCS algorithm^55^. We did the energy minimization of the entire system and then gradually increased the system’s temperature using the V-rescale thermostat with position restraints for heavy atoms in the protein. A 5-ns production run with a time step of 2 fs in the NPT ensemble was performed with position restraints for backbone atoms in the protein to relax the side-chain conformations. The temperature and pressure of the system were controlled at 310 K and 1 bar using a Nose-Hoover thermostat^56^,^57^ and Parrinello-Rahman barostat^58^,^59^, respectively.

## Additional Information

**How to cite this article**: Fujiwara, Y. *et al.* Structural basis for the membrane association of ankyrinG via palmitoylation. *Sci. Rep.* **6**, 23981; doi: 10.1038/srep23981 (2016).

## Supplementary Material

Supplementary Information

## Figures and Tables

**Table 1:** Data collection and refinement statistics.

	ANK3 (R1-R5), Reduced	ANK3 (R1-R5), Oxidized
**Data collection**		
Space group	*P*2_1_2_1_2	*C*121
Cell dimensions		
a, b, c (Å)	96.01, 102.54, 24.37	137.18, 67.55, 44.283
a, b, g (°)	90.00, 90.00, 90.00	90.00, 99.88, 90.00
Resolution (A)	50.0–1.62 (1.65–1.62)	50.0–1.83 (1.86–1.83)
*R*_sym_	8.1 (36.0)	7.1 (78.7)
*I*/σ*I*	31.9 (5.5)	28.7 (2.1)
Completeness (%)	99.9 (99.5)	98.3 (97.5)
Redundancy	6.7 (6.2)	4.0 (3.8)
**Refinement**		
Resolution (Å)	50.0–1.62	50.0–1.83
No. reflections	29,921	32,867
*R*_work_/*R*_free_	16.4/20.6	18.5/22.1
No. atoms		
protein	2,382	1,986
water	238	127
ligand	32	45
average B-factors		
protein	13.4	37.1
water	22.7	37.2
ligand	23.2	62.3
R.m.s. deviations		
Bond length (Å)	0.024	0.022
Bond angles (°)	1.91	1.82

Values in parentheses are for the highest-resolution shell.
