# Supplementary material for: Structural basis for the membrane association of ankyrinG via palmitoylation
Source: Sci Rep. 2016 Apr 5;6:23981. doi: 10.1038/srep23981 (PMC4820748; doi:10.1038/srep23981)
Supplement: Supplementary Information [file srep23981-s1.pdf]

SUPPLEMENTARY INFORMATION

Structural basis for the membrane association of ankyrinG via palmitoylation

Yuichiro Fujiwara , Hiroko X. Kondo, Matsuyuki Shirota, Megumi Kobayashi1, Kohei Takeshita, Atsushi Nakagawa, Yasushi Okamura & Kengo Kinoshita

Supplementary Figures S1-S6

SUPPLEMENTARY FIGURES

Fig. S1

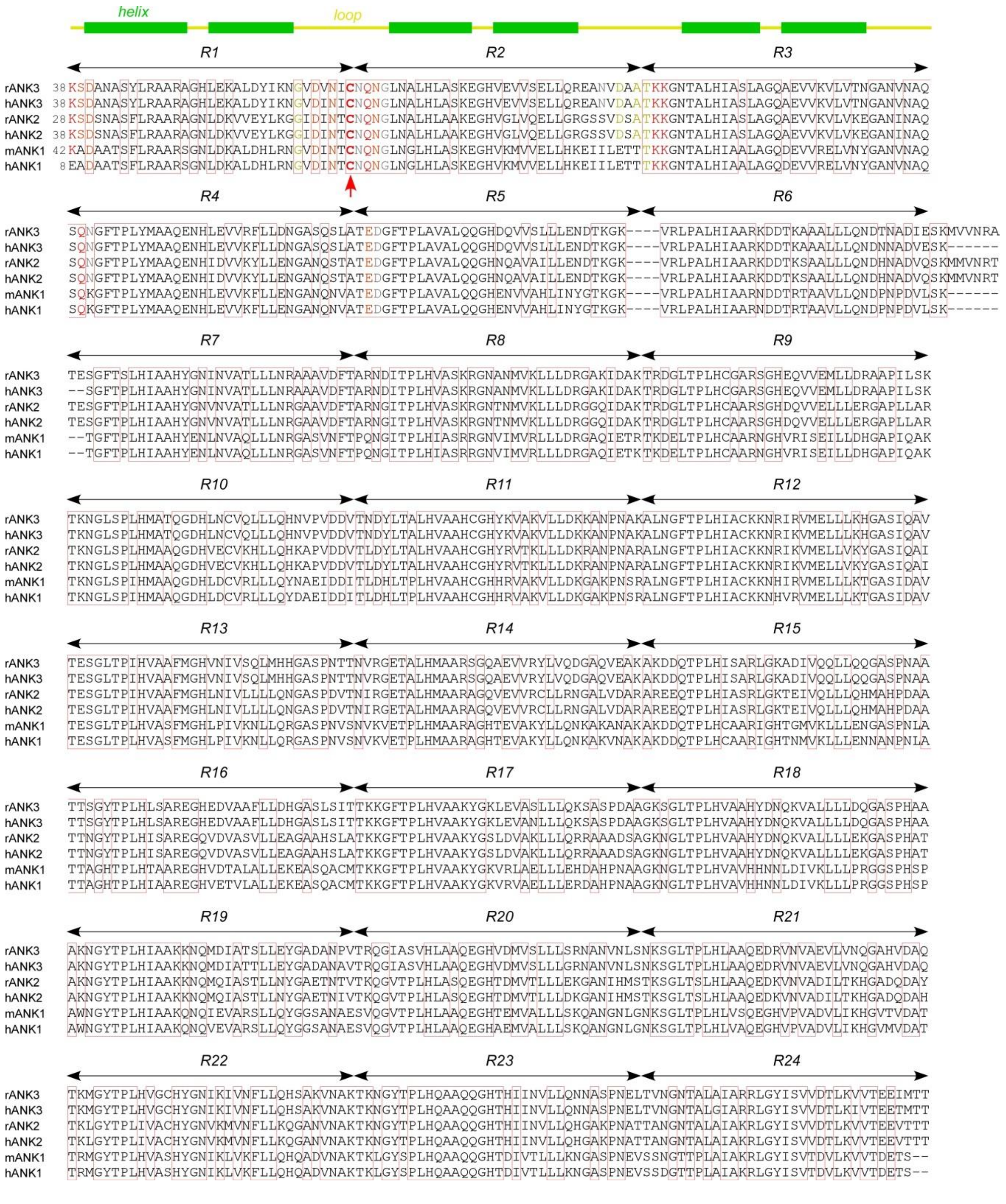

Amino acid alignment of the membrane-binding domain of Ankyrin family proteins

Twnenty-four ankyrin repeats (R1-R24) comprise the membrane-binding domain of ankyrin (270 kDa) family proteins. Exactly 33 amino acid residues are aligned in each repeat. An exceptionally short loop is seen between R5 and R6 and there is an extra sequence between R6 and R7. Secondary structure is shown at the top. An arrow indicates the position of Cys70. Residues that interact to the membrane (Fig. 5) are highlighted in color. Conserved residues are boxed. **Protein abbreviations** are **rANK3** (rat AnkG, AF102552.1); **hANK3** (human AnkG, NM_001149); **rANK2** (rat AnkB, XM_00107608); **hANK2** (human AnkB, NM_001148); **mANK1** (mouse AnkR, NP_001104253); **hANK1** (human AnkR, NP_000028).

Fig. S2

```
Ankyrin Repeat ←-----------Repeat 1------------→←----------Repeat 2------------→
Structure---------- --Helix_1a---------1b---------Loop-----------2a---------2b------------------------------

ANK1-----MPYSVGFREADAATSFLRAARSGNLDKALDHLRNGVDINTCNQNGLNGLHLASKEGHVKMVVELLHKEIILETTTKKGNTALHIAALAG
ANK2-----SQRRKRPKKSDSNASFLRAARAGNLDKVVEYLKGGIDINTCNQNGLNALHLAAKEGHVGLVQELLGRGSSVDSATKKGNTALHIASLAG
ANK3-----KRSRDRKKKSDANASYLRAARAGHLEKALDYIKNGVDINICNQNGLNALHLASKEGHVEVVSELLQREANVDAATKKGNTALHIASLAG
ABTB1-------------MDTSDLFASCRKGDVGRVRYLLEQRDVEVNVRDKWDSTPLYYACLCGHEELVLYLLANGARCEANTFDGERCLYGALSDP
ANKFY1---AQLLYKMIKSKTEYPLHKAIKVEREDVVFLYLIEMDSQLPGKLNEADHNGDLALDLALSRRLESIATTLVSHKADVDMVDKSGWSLLHK
ANKHD1---KAENSHNAGQVDTRSLAEACSDGDVNAVRKLLDEGRSVNEHTEEGESLLCLACSAGYYELAQVLLAMHANVEDRGNKGDITPLMAASSG
ANKK1----PRDEELCIYENKVTPLHFLVAQGSVEQVRLLLAHEVDVDCQTASGYTPLLIAAQDQQPDLCALLLAHGADANRVDEDGWAPLHFAAQNG
ANKRD1---DKNNPDVCDEYKRTALHRACLEGHLAIVEKLMEAGAQIEFRDMLESTAIHWASRGGNLDVLKLLLNKGAKISARDKLLSTALHVAVRTG
ANKS1A---RGPNVNCVDSTGYTPLHHAALNGHKDVVEVLLRNDALTNVADSKGCYPLHLAAWKGDAQIVRLLIHQGPSHTRVNEQNNDNETALHCAA
ANKZF1---LDEAKAPGQPELWNALLAACRAGDVGVLKLQLAPSPADPRVLSLLSAPLGSGGFTLLHAAAAAGRGSVVRLLLEAGADPTVQDSRARPP
ASB1-----EQFCDHPLEHCEDTRLHDAAYVGDLQTLRSLLQEESYRSRINEKSVWCCGWLPCTPLRIAATAGHGSCVDFLIRKGAEVDLVDVKGQTA
ASZ1-----QKLKRLLPIEEKKEKFKKAMTIGDVSLVQELLDSGISVDSNFQYGWTPLMYAASVANAELVRVLLDRGANASFEKDKQSILITACSAHG
BARD1----LPNMAVKRNHRGETLLHIASIKGDIPSVEYLLQNGSDPNVKDHAGWTPLHEACNHGHLKVVELLLQHKALVNTTGYQNDSPLHDAAKNG
BAT4-----GQGRSLEAEDKMTHRILRAAQEGDLPELRRLLEPHEAGGAGGNINARDAFWWTPLMCAARAGQGAAVSYLLGRGAAWVGVCELSGRDAA
BCL3-----DIAMATRADEDGDTPLHIAVVQGNLPAVHRLVNLFQQGGRELDIYNNLRQTPLHLAVITTLPSVVRLLVTAGASPMALDRHGQTAAHLA
CAMTA1---SKHLIHSKTFRGMTLLHLAAAQGYATLIQTLIKWRTKHADSIDLELEVDPLNVDHFSCTPLMWACALGHLEAAVVLYKWDRRAISIPDS
CDKN2A---MEPAAGSSMEPSADWLATAAARGRVEEVRALLEAGALPNAPNSYGRRPIQVMMMGSARVAELLLLHGAEPNCADPATLTRPVHDAAREG
CENTB1---ADVNWVNGGQDNATPLIQATAANSLLACEFLLQNGANVNQADSAGRGPLHHATILGHTGLACLFLKRGADLGARDSEGRDPLTIAMETA
CENTG1---GPLDTSVEDPQLRSPLHLAAELAHVVITQLLLWYGADVAARDAQGRTALFYARQAGSQLCADILLQHGCPGEGGSAATTPSAATTPSIT
CLPB-----VVHCYSKSPSNKDAALLEAARANNMQEVSRLLSEGADVNAKHRLGWTALMVAAINRNNSVVQVLLAAGADPNLGDDFSSVYKTAKEQGI
CTTNBP2--LMSGGPAPLAGRPTLLQQAAAQGNVTLLSMLLNEEGLDINYSCEDGHSALYSAAKNGHTDCVRLLLSAEAQVNAADKNGFTPLCAAAAQ
DAPK1----SNYDVNQPNKHGTPPLLIAAGCGNIQILQLLIKRGSRIDVQDKGGSNAVYWAARHGHVDTLKFLSENKCPLDVKDKSGEMALHVAARYG
DGKZ-----AGGDLMHRDEQSRTLLHHAVSTGSKDVVRYLLDHAPPEILDAVEENGETCLHQAAALGQRTICHYIVEAGASLMKTDQQGDTPRQRAEK
EHMT1----LDSEKPKKLRFHPKQLYFSARQGELQKVLLMLVDGIDPNFKMEHQNKRSPLHAAAEAGHVDICHMLVQAGANIDTCSEDQRTPLMEAAE
GABPB2---------MSLVDLGKKLLEAARAGQDDEVRILMANGAPFTTDWLGTSPLHLAAQYGHYSTTEVLLRAGVSRDARTKVDRTPLHMAASEGH
GIT1-----CRDDDGVTAKDLSKQLHSSVRTGNLETCLRLLSLGAQANFFHPEKGTTPLHVAAKAGQTLQAELLVVYGADPGSPDVNGRTPIDYARQA
ILK------------------MDDIFTQCREGNAVAVRLWLDNTENDLNQGDDHGFSPLHWACREGRSAVVEMLIMRGARINVMNRGDDTPLHLAASH
INVS-----KSENLLFAGSSLASQVHAAAVNGDKGALQRLIVGNSALKDKEDQFGRTPLMYCVLADRLDCADALLKAGADVNKTDHSQRTALHLAAQK
KRIT1----SVTEDKERQWVDDFPLHRSACEGDSELLSRLLSERFSVNQLDSDHWAPIHYACWYGKVEATRILLEKGKCNPNLLNGQLSSPLHFAAGG
LRRK1----EACDQCASQLEKGQLLSIPAAYGDLEMVRYLLSKRLVELPTEPTDDNPAVVAAYFGHTAVVQELLESLPGPCSPQRLLNWMLALACQRG
MIB1-----KKLFETQESGDLNEELVKAAANGDVAKVEDLLKRPDVDVNGQCAGHTAMQAASQNGHVDILKLLLKQNVDVEAEDKDGDRAVHHAAFGD
MPHOSPH8-EEYNLDQEDSSGMTLVMLAAAGGQDDLLRLLITKGAKVNGRQKNGTTALIHAAEKNFLTTVAILLEAGAFVNVQQSNGETALMKACKRG
CDKN2C----------MAEPWGNELASAAARGDLEQLTSLLQNNVNVNAQNGFGRTALQVMKLGNPEIARRLLLRGANPDLKDRTGFAVIHDAARAGF
MTPN---------------MCDKEFMWALKNGDLDEVKDYVAKGEDVNRTLEGGRKPLHYAADCGQLEILEFLLLKGADINAPDKHHITPLLSAVYEG
NFKB1----QRHLTAVQDENGDSVLHLAIIHLHSQLVRDLLEVTSGLISDDIINMRNDLYQTPLHLAVITKQEDVVEDLLRAGADLSLLDRLGNSVLH
NFKBIA---SEPWKQQLTEDGDSFLHLAIIHEEKALTMEVIRQVKGDLAFLNFQNNLQQTPLHLAVITNQPEIAEALLGAGCDPELRDFRGNTPLHLA
NOTCH1---SLHNQTDRTGETALHLAARYSRSDAAKRLLEASADANIQDNMGRTPLHAAVSADAQGVFQILIRNRATDLDARMHDGTTPLILAARLAV
NUDT12----SSVKRSLKQEIVTQFHCSAAEGDIAKLTGILSHSPSLLNETSENGWTALMYAARNGHPEIVQFLLEKGCDRSIVNKSRQTALDIAVFW
OSBPL1A---NCKGRSKSNLGWTPLHLACYFGHRQVVQDLLKAGAEVNVLNDMGDTPLHRAAFTGRKELVMLLLEYNADTTIVNGSGQTAKEVTHAEE
PLA2G6----SCANCAENEEGCTPLHLACRKGDGEILVELVQYCHTQMDVTDYKGETVFHYAVQGDNSQVLQLLGRNAVAGLNQVNNQGLTPLHLACQ
PPP1R12A--KRQKTKVKFDDGAVFLAACSSGDTDEVLKLLHRGADINYANVDGLTALHQACIDDNVDMVKFLVENGANINQPDNEGWIPLHAAASCG
PSMD10---------MEGCVSNLMVCNLAYSGKLEELKESILADKSLATRTDQDSRTALHWACSAGHTEIVEFLLQLGVPVNDKDDAGWSPLHIAASA
RAI14-----RKSDTNEWNKNDDRLLQAVENGDAEKVASLLGKKGASATKHDSEGKTAFHLAAAKGHVECLRVMITHGVDVTAQDTTGHSALHLAAKN
RFXANK----NEVSALPATLDSLSIHQLAAQGELDQLKEHLRKGDNLVNKPDERGFTPLIWASAFGEIETVRFLLEWGADPHILAKERESALSLASTG
RIPK4-----PQDVDLALDSGASLLHLAVEAGQEECAKWLLLNNANPNLSNRRGSTPLHMAVERRVRGVVELLLARKISVNAKDEDQWTALHFAAQNG
RNASEL----TSSSGRRAAVEDNHLLIKAVQNEDVDLVQQLLEGGANVNFQEEEGGWTPLHNAVQMSREDIVELLLRHGADPVLRKKNGATPFILAAI
TNKS------NVNAKDMAGRKSSPLHFAAGFGRKDVVEHLLQMGANVHARDDGGLIPLHNACSFGHAEVVSLLLCQGADPNARDNWNYTPLHEAAIKG
TP53BP2---VDDPSLPNDEGITALHNAVCAGHTEIVKFLVQFGVNVNAADSDGWTPLHCAASCNNVQVCKFLVESGAAVFAMTYSDMQTAADKCEEM
TRPA1-----QKKLKRCDDMDTFFLHYAAAEGQIELMEKITRDSSLEVLHEMDDYGNTPLHCAVEKNQIESVKFLLSRGANPNLRNFNMMAPLHIAVQ
TRPC3-----FNDRGTSLTAEEERFLDAAEYGNIPVVRKMLEESKTLNVNCVDYMGQNALQLAVGNEHLEVTELLLKKENLARIGDALLLAISKGYVR
TRPV1-----ASTEKTLRLYDRRSIFEAVAQNNCQDLESLLLFLQKSKKHLTDNEFKDPETGKTCLLKAMLNLHDGQNTTIPLLLEIARQTDSLKELV
ZDHHC17---GYDVRQPDKENVTLLHWAAINNRIDLVKYYISKGAIVDQLGGDLNSTPLHWATRQGHLSMVVQLMKYGADPSLIDGEGCSCIHLAAQF
```

Comparison of the amino acid sequence of the palmitoylation loop among ankyrin repeat domain proteins

Multiple alignments of the R1 and R2 ankyrin repeat domains are shown. Secondary structures were determined from the crystal structure of ANK3 (R1-5). Cys (colored in red) situated in the linker (yellow) between the first and second repeats (green) is highlighted. **Protein abbreviations** (protein name, total number of the ankyrin repeat, NCBI Reference Sequence) are **ANK1** (Ankyrin 1, 24 repeats, NP_000028); **ANK2** (Ankyrin 2, 24 repeats, NM_001148); **ANK3** (Ankyrin 3, 24 repeats, NM_001149); **ABTB1** (Ankyrin repeat and BTB/POZ domain-containing protein 1, 2 repeats, NM_032548); **ANKFY1** (Rabankyrin-5, 21 repeats, NM_001257999) ; **ANKHD1**(Ankyrin repeat and KH domain-containing protein 1, 25 repeats, NM_001197030) ;**ANKK1**(Ankyrin repeat and protein kinase domain-containing protein 1, 12 repeats, NM_178510) ; **ANKRD1**(Ankyrin repeat domain-containing protein 1, 5 repeats, NM_014391); **ANKS1A**(Ankyrin repeat and SAM domain-containing protein 1A, 6 repeats, NM_015245) ; **ANKZF1**(Ankyrin repeat and zinc finger domain-containing protein 1, 2 repeats, NM_001042410);**ASB1** (Ankyrin repeat and SOCS box protein 1, 6 repeats, NM_001040445); **ASZ1** (Ankyrin repeat, SAM and basic leucine zipper domain-containing protein 1, 6 repeats, NM_130768) ;**BARD1** (BRCA1-associated RING domain protein 1, 4 repeats, NM_000465); **BAT4** (G patch domain and ankyrin repeat-containing protein 1, 2 repeats, NM_001199237); **BCL3**(B-cell lymphoma 3 protein, 7 repeats, NM_005178); **CAMTA1**(Calmodulin-binding transcription activator 1, 3 repeats, NM_001195563); **CDKN2A**(Cyclin-dependent kinase inhibitor 2A, isoforms 1/2/3, 4 repeats, NM_000077); **CENTB1**(Arf-GAP with coiled-coil, ANK repeat and PH domain-containing protein 1,3 repeats, NM_014716);**CENTG1**(Arf-GAP with GTPase, ANK repeat and PH domain-containing protein 2,2

repeats, NM_001122772); **CLPB**(Caseinolytic peptidase B protein homolog,4 repeats, NM_001258392); **CTTNBP2**(Cortactin-binding protein 2, 6 repeats, NM_033427); **DAPK1**(Death-associated protein kinase 1,10 repeats,NM_001288729); **DGKZ**(Diacylglycerol kinase zeta,2 repeats,NM_001105540); **EHMT1**(Histone-lysine N-methyltransferase EHMT1,8 repeats ,NM_001145527); **GABPB2**(GA-binding protein subunit beta-1,5 repeats ,NM_002041); **GIT1**(ARF GTPase-activating protein GIT1,3 repeats, NM_001085454);**ILK**(Integrin-linked protein kinase,5 repeats, NM_001014794); **INVS**(Inversin,16 repeats,NM_014425); **KRIT1**(Krev interaction trapped protein 1,4 repeats,NM_0010134069); **LRRK1**(Leucine-rich repeat serine/threonine-protein kinase 1,13 repeats,NM_024652); **MIB1**(E3 ubiquitin-protein ligase MIB1,9 repeats,NM_020774); **MPHOSPH8**(M-phase phosphoprotein 8,4 repeats,NM_017520) ;CDKN2C(Cyclin-dependent kinase 4 inhibitor C,4 repeats,NM_001262);**MTPN**(Myotrophin,3 repeats,NM_145808); **NFKB1**(Nuclear factor NF-kappa-B p105 subunit,7 repeats,NM_001165412); **NFKBIA**(NF-kappa-B inhibitor alpha,5 repeats,NM_020529); **NOTCH1**(Neurogenic locus notch homolog protein 1,5 repeats,NM_017617); **NUDT12**(Peroxisomal NADH pyrophosphatase NUDT12,3 repeats,NM_001300741); **OSBPL1A**(Oxysterol-binding protein-related protein 1,3 repeats, NM_001242508) ;**PLA2G6**(85/88 kDa calcium-independent phospholipase A2,7 repeats,NM_001004426) ; **PPP1R12A**(Protein phosphatase 1 regulatory subunit 12A,6 repeats, NM_001143885); **PSMD10**(26S proteasome non-ATPase regulatory subunit 10,7 repeats,NM_002814); **RAI14**(Ankycorbin ,7 repeats,NM_001145520); **RFXANK**(DNA-binding protein RFXANK ,5 repeats,NM_001278727); **RIPK4**(Receptor-interacting serine/threonine-protein kinase 4 ,10 repeats,NM_020639); **RNASEL**(2-5A-dependent

ribonuclease,9 repeats, NM_021133); TNKS(Tankyrase-1,15 repeats,NM_003747); **TP53BP2**(Apoptosis-stimulating of p53 protein 2,2 repeats,NM_001031685); **TRPA1**(Transient receptor potential cation channel subfamily A member 1,15 repeats, NM_007332); **TRPC3**(Short transient receptor potential channel 3,4 repeats,NM_001130698); **TRPV1**(Transient receptor potential cation channel subfamily V member 1,6 repeats,NM_018727); **ZDHHC17**(Palmitoyltransferase ZDHHC17,5 repeats,NM_015336)

Fig. S3

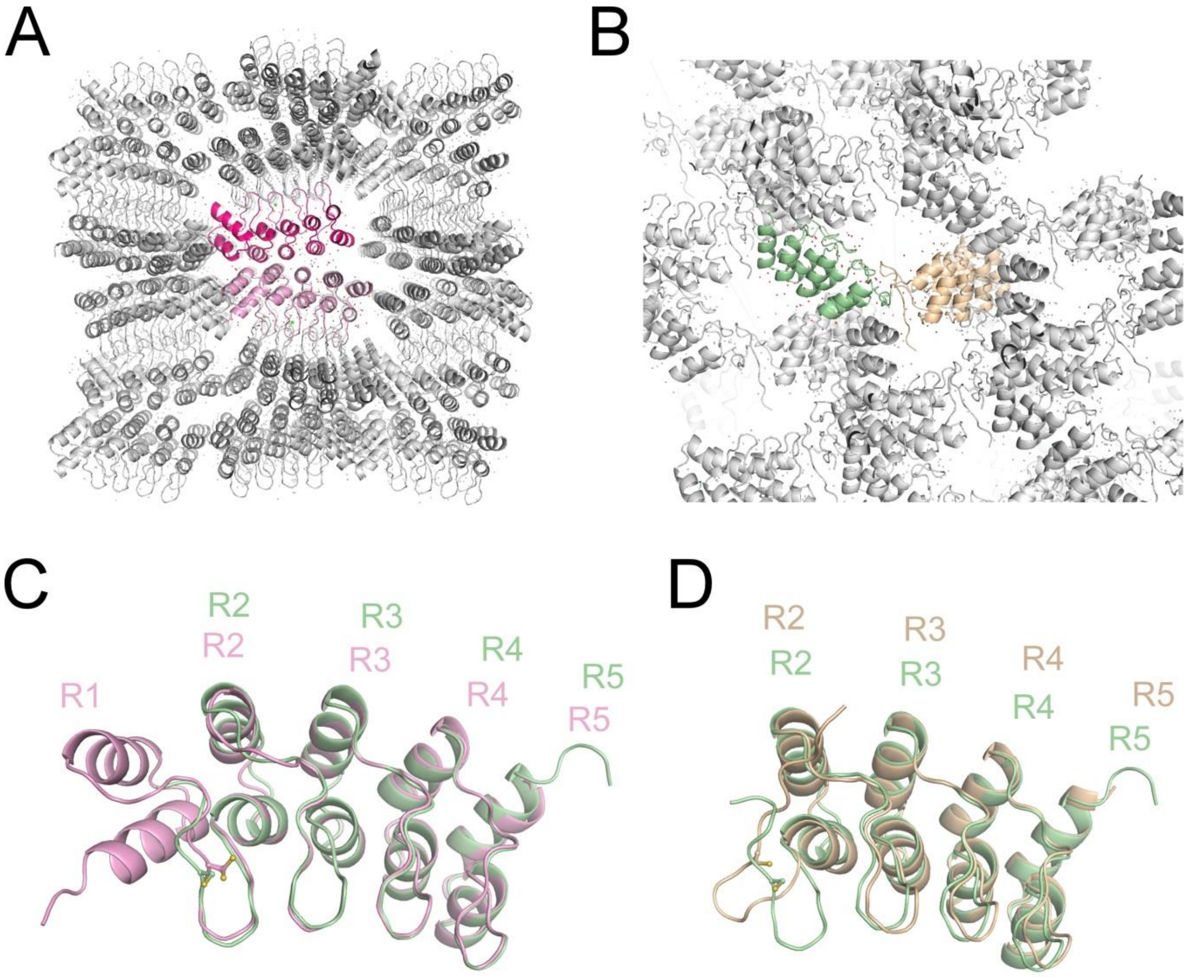

Comparison of the structures of the reduced and oxidized forms of AnkG

A) The crystal packing of the $P2_12_12$ crystal (the reduced form). Two molecules of AnkG (R1-R5) are in the asymmetric unit. B) The crystal packing of the C121 crystal (the oxidized form). An AnkG (R1-R5) dimer is in the asymmetric unit. **C)** Superposition of the reduced and oxidized structures (overall Cα-r.m.s.d. of 0.732 Å). Regarding the finger loop of R2, the Ca^{2+} binding pocket, there is little difference between reduced and oxidized structures (Cα-r.m.s.d. of 0.601 Å). The structure of the R2 finger loop is also close to those of AnkB (Cα-r.m.s.d.s of 0.497 Å;

AnkG-oxidized vs. AnkB-Nav, 0.375 Å; AnkG-oxidized vs. AnkB-AS). **D)** Superposition of each monomer in the crystal form of the oxidized dimer (overall Cα- r.m.s.d. of 1.753 Å).

Fig. S4

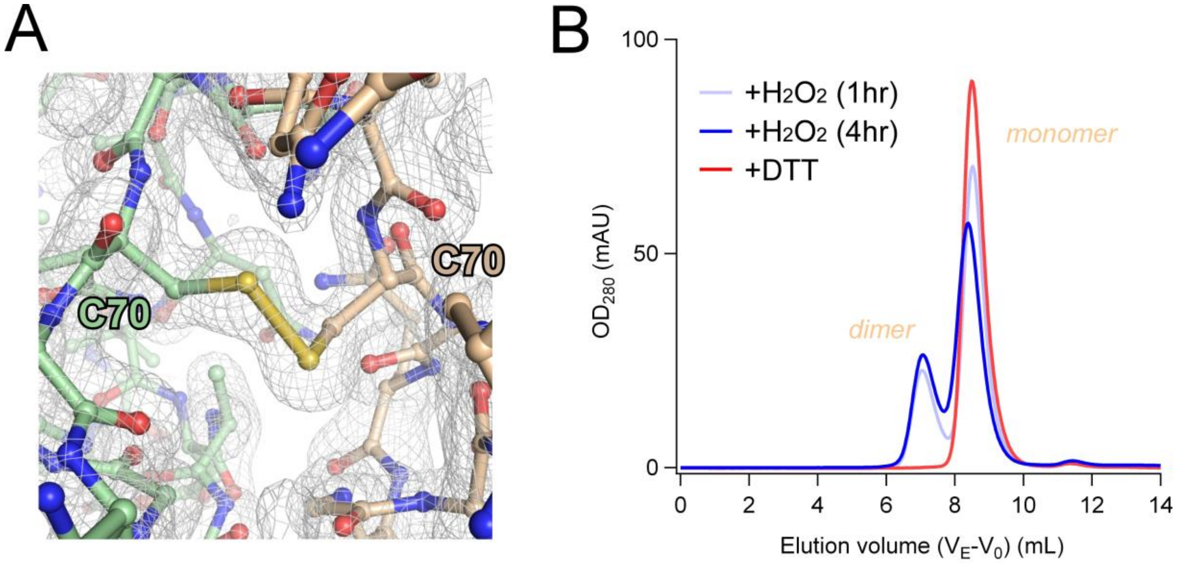

A) Structure (stick models) and 2Fo-Fc maps (1.5 σ) around Cys70. **B)** AnkG's ability to form a disulfide bond in solution was examined. Molecular stoichiometry determined using size exclusion chromatography monitored at 280 nm. The dimer population increased progressively when the protein sample was left in mildly oxidizing buffer (100 μM H_2O_2), and reduction of the protein using 5mM DTT led to recovery of the monomeric form. The elution volume V_E was corrected for the void elution volume by subtracting the volume of blue dextran (V_0).

Fig. S5

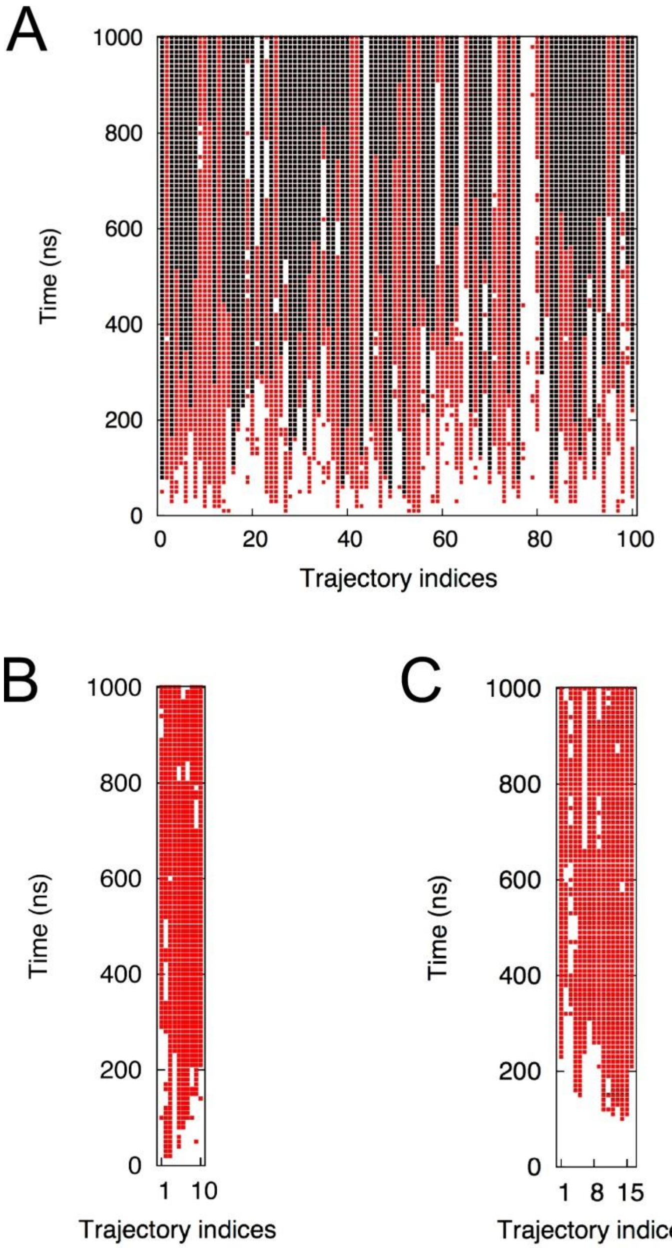

Data in each trajectory of the MD simulation for the membrane association of AnkG

A) Membrane contacts (red) and insertions (black) in the simulation of palmitoylated AnkG (Fig. 3). Events in all 100 trajectories were counted in chronological order. **B,C)** Membrane contact events by the non-palmitoylated AnkG (Fig. 4A) (**B**) and the AnkG dimer (Fig. 4B) (**C**).

Fig S6

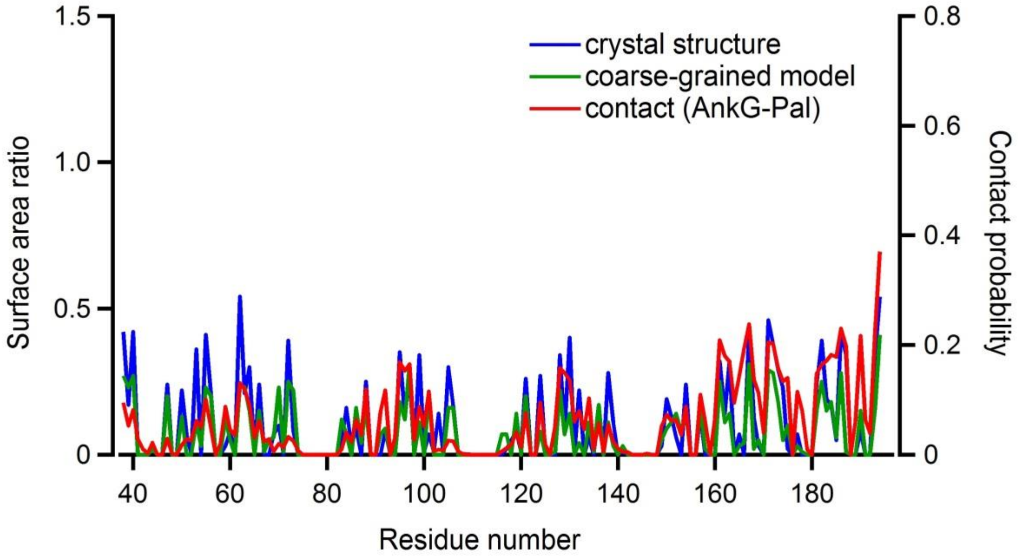

Correlations between contact probability and surface ratio.

Surface area ratios were calculated for the AnkG crystal structure and for the coarse-grained model. Data were plotted over the contact probability of the palmitoylated AnkG (Fig. 3C). Distributions nicely overlapped with each other, suggesting that AnkG contacted the membrane with its surface randomly before anchoring.
